# PKC-epsilon deficiency alters progenitor cell populations in favor of megakaryopoiesis

**DOI:** 10.1371/journal.pone.0182867

**Published:** 2017-08-04

**Authors:** John C. Kostyak, Elisabetta Liverani, Satya P. Kunapuli

**Affiliations:** 1 Sol Sherry Thrombosis Research Center, Temple University Lewis Katz School of Medicine, Philadelphia, Pennsylvania, United States of America; 2 Center for Inflammation, Translational and Clinical Lung Research, Temple University Lewis Katz School of Medicine, Philadelphia, Pennsylvania, United States of America; 3 Department of Pharmacology, Temple University Lewis Katz School of Medicine, Philadelphia, Pennsylvania, United States of America; 4 Department of Physiology, Temple University Lewis Katz School of Medicine, Philadelphia, Pennsylvania, United States of America; Ludwig-Maximilians-Universitat Munchen, GERMANY

## Abstract

**Background:**

It has long been postulated that Protein Kinase C (PKC) is an important regulator of megakaryopoiesis. Recent contributions to the literature have outlined the functions of several individual PKC isoforms with regard to megakaryocyte differentiation and platelet production. However, the exact role of PKCε remains elusive.

**Objective:**

To delineate the role of PKCε in megakaryopoiesis.

**Approach and results:**

We used a PKCε knockout mouse model to examine the effect of PKCε deficiency on platelet mass, megakaryocyte mass, and bone marrow progenitor cell distribution. We also investigated platelet recovery in PKCε null mice and TPO-mediated signaling in PKCε null megakaryocytes. PKCε null mice have higher platelet counts due to increased platelet production compared to WT littermate controls (p<0.05, n = 8). Furthermore, PKCε null mice have more bone marrow megakaryocyte progenitor cells than WT littermate control mice. Additionally, thrombopoietin-mediated signaling is perturbed in PKCε null mice as Akt and ERK1/2 phosphorylation are enhanced in PKCε null megakaryocytes stimulated with thrombopoietin. Finally, in response to immune-induced thrombocytopenia, PKCε null mice recovered faster and had higher rebound thrombocytosis than WT littermate control mice.

**Conclusions:**

Enhanced platelet recovery could be due to an increase in megakaryocyte progenitor cells found in PKCε null mice as well as enhanced thrombopoietin-mediated signaling observed in PKCε deficient megakaryocytes. These data suggest that PKCε is a negative regulator of megakaryopoiesis.

## Introduction

Mature megakaryocytes are large (up to 100μM), polyploid cells that reside in bone marrow [1]. Megakaryocytes develop from hematopoietic stem cells (HSC) through stimulation by the cytokine thrombopoietin (TPO). TPO binds its receptor c-Mpl and induces a signaling cascade that causes HSCs to differentiate into megakaryocytes. Although other cytokines such as stem cell factor [2], Interleukin-6 [3], and Interleukin-11 [4] are all important for megakaryopoiesis, TPO is widely considered the primary regulator of megakaryocyte differentiation and platelet production *in vivo*.

TPO binds its receptor (c-Mpl) expressed on HSCs and elicits a complex cell signaling mechanism. Upon TPO binding its receptor the janus kinase, JAK2 is activated. JAK2 is required for TPO signaling and active JAK2 results in the phosphorylation of several other factors including but not limited to, Akt, ERK1/2, and p38MAPK [5–9]. Additionally, TPO engagement results in the activation of the Src family kinase, Lyn which may serve as a negative feedback mechanism, since Lyn-null mice display significant increases in megakaryopoiesis [10]. Additionally, TPO can induce activation of FAK without FAK integrin engagement, perhaps via CIB1 [11, 12]. FAK-null mice also display increased megakaryopoiesis as well as greatly reduced Lyn kinase activity [11]. This suggests that FAK may be integral as a mediator of megakaryopoiesis by activating Lyn kinase. Additionally, it was recently reported that one residue on c-Mpl (Y591) acts in a negative capacity and binds spleen tyrosine kinsase (Syk), Bruton’s tyrosine kinase (BTK), and src homology region 2 domain-containing phosphatase-1 (SHP-1) [13]. The above factors all contribute to megakaryocyte differentiation, which culminates in platelet production. Although recent experimental evidence has enhanced our knowledge of megakaryopoiesis, the exact mechanisms regulating megakaryocyte differentiation and platelet production remain elusive. PKC’s play a vital role in the differentiation of megakaryoblastic cell lines and may be integral for primary megakaryocyte differentiation as well.

PKC’s are serine/threonine kinases that have a wide range of functions in a variety of different cell types. They are classified in one of three categories based on their cofactor requirements: conventional, novel, and atypical. Conventional (α, β, β, γ) PKC’s respond to calcium and phorbol esters, while novel (θ, δ, η, ε) PKC’s lack the conserved region required to respond to calcium.

Several PKC isoforms play important roles in platelet function. We have previously reported that PKCθ positively regulates platelet functional responses by regulating GPVI and PAR receptors [14]. PKCδ also positively regulates PAR-mediated platelet function, but unlike PKCθ, PKCδ negatively regulates GPVI-mediated platelet functional responses, perhaps through interaction with the Src-family kinase Lyn and SH_2_-containing inositol phosphatase-1 (SHIP-1) [15, 16]. Another novel PKC isoform, PKCε, is a negative regulator of PAR-mediated functional responses and ADP-induced thromboxane generation [17]. PKCα regulates granular secretion in platelets via an interaction with glycogen-synthase-kinase 3β (GSK3β) [18]. Furthermore, PKCα and PKCβ are positive regulators of thrombus formation in zebrafish [19].

While much is known regarding PKC function in platelets, the role of PKC’s in primary megakaryocyte differentiation has been largely unexplored. Oshevski et al., revealed that mRNA expression of PKCα, PKCβI/II, PKCθ, and PKCδ were enhanced in human megakaryocytes compared to progenitor cells, while PKCε mRNA expression remained unchanged [20]. However, it was later revealed that PKCε expression increases in the early stages of human megakaryocyte differentiation, but is downmodulated as megakaryocytes reach maturity [21].

In this manuscript we show, using a knockout mouse model, that PKCε is a negative regulator of megakaryocyte differentiation and platelet production. PKCε deficient mice have an increased platelet mass due to increased platelet production. We also observed that PKCε deficient mice have a higher percentage of bone marrow megakaryocyte progenitor cells than littermate control WT mice. Finally, PKCε null mice recovery faster from immune-induced thrombocytopenia than WT control mice, which may be due to altered TPO-mediated signaling.

## Material and methods

### Antibodies and reagents

All reagents were purchased from Sigma-Aldrich unless otherwise noted. Cell culture reagents including Iscove’s Modified Dulbecco’s Medium (IMDM), heat-inactivated fetal bovine serum (FBS), and penicillin/streptomycin (P/S) were purchased from Thermo Fisher (Pittsburgh, PA). Monoclonal antibodies against Akt, ERK1/2, FAK, JAK2, PKCδ, PKCε, and PKCα were purchased from Santa Cruz Biotechnologies (Santa Cruz, CA). Polyclonal antibodies against b-actin, phospho-ERK1/2, phospho-Akt S473, phospho-FAK Y925, and phopho-JAK2 were purchased from Cell Signaling Technologies (Danvers, MA). FITC-labeled anti-mouse CD41 antibody and a purified anti-mouse CD41 antibody were purchased from BD Biosciences (San Jose, CA). All antibodies used for progenitor cell analysis were from eBiosciences (San Diego, CA).

### Animals

PKCε^-/-^ mice were a kind gift from Dr. Robert Messing (Gallo Centre, San Francisco, CA). All animal protocols were approved by the Temple University Institutional Animal Care and Use Committee (protocol #4408) and carried out in accordance with the Guide for the Care and Use of Laboratory Animals of the National Institutes of Health.

### Blood cell enumeration

Blood was collected from PKCε^-/-^ and littermate control mice via cardiac puncture. Anti-coagulated blood was analyzed by a Hemavet (Drew Scientific Inc., Waterbury, CT) blood cell analyzer.

### Platelet production assay

Platelet production was assessed as previously described [12]. Briefly, 1mL of blood from PKCε^-/-^ or littermate control mice was added to 60mL of 2mM EDTA in PBS containing 10 mg/mL Thiazole Orange and incubated for 30 minutes at room temperature. The samples were then fixed in 1% formalin for 15 minutes at room temperature and analyzed by flow cytometry using an LSRII flow cytometer and FACSDiva software (BD Biosciences). Thiazole Orange positive platelets were considered “new”.

### Platelet clearance assay

Platelet Clearance was assessed as previously described [12]. Briefly, PKCε^-/-^ and WT littermate control mice were injected I.V. with 35 mg/g sulfo-NHS-biotin (Pierce Chemical). Using a submandibular method, 30 μL blood was collected 3 hours after I.V. injection and then every 24 hours for 4 days. Anti-coagulated blood was diluted 20X in PBS and incubated with 10 mg/mL Thiazole Orange and Streptavidin-PE antibody (BD Biosciences) for 30 minutes at 4C. The samples were then fixed and analyzed via flow cytometry using an LSRII flow cytometer and FACSDiva software.

### Megakaryocyte analysis

Megakaryocytes were analyzed via flow cytometry as previously described [12]. Bone marrow femurs and tibiae, or spleens from PKCε^-/-^ or WT littermate control mice were flushed into IMDM and passed through a 22-gauge needle to create a single-cell suspension. Cells were then centrifuged at 340 X g for 3 minutes and resuspended in ACK buffer (0.15 M NH4Cl, 10 mM KHCO3, 0.1 mM Na2EDTA, pH 7.4) for 5 minutes to lyse the red cells. In case of cell culture, the cells were washed in PBS then resuspended in IMDM containing FBS, P/S, and 50 ng/mL recombinant mouse TPO (Peprotech) prior to incubation for 5 days. For flow cytometric analysis, 2 X 10^6^ cultured cells or bone marrow cells (after red cell lysis) were washed in PBS and resuspended in PBS containing 2 mM EDTA and labeled with FITC-CD41 antibody for 30 minutes at 4°C. The cells were then washed in PBS and fixed in 0.5% formalin for 20 minutes at room temperature. After washing in PBS the cells were permeabilized in 70% methanol for 60 minutes at 4°C, followed by another wash and treatment with 10 mg/mL RNAse A for 30 minutes at 37°C. Finally, the cells were treated with 10 μg/mL propidium iodide and analyzed using an LSRII flow cytometer and FACSdiva software.

### Progenitor cell analysis

Bone marrow progenitor cell analysis, including gating and overall scheme, was performed as previously described [22]. A detailed schematic of the gating scheme used was previously described by Pronk et al [23]. Following isolation of red cell deficient bone marrow, 10^6^ cells were labeled with collection of antibodies (Hematopoietic lineage cocktail tagged with e450, APC-c-Kit, PE-Sca-1, FITC-CD150, and either PECy7-CD105 or PECy7-CD41) for 30 minutes at 4°C. Cells were then fixed in 1% formalin and analyzed using an LSRII flow cytometer and FACSdiva software.

### Immune-induced thrombocytopenia

Experiments to analyze recovery from thrombocytopenia were carried out as previously described [24]. Briefly, baseline platelet counts were taken from 10–12 week-old PKCε^-/-^ or WT littermate control mice 5 days prior to treatment with 50 μg/kg anti-mouse CD41 antibody via I.P. injection. Blood cell counts were then taken every 24 hours after I.P. injection via submandibular puncture for 5 days and again at 7 days.

### Proplatelet production

Bone marrow isolated from PKCε^+/+^ and PKCε^-/-^ mice was cultured as described in “Megakaryocyte Analysis” to expand the megakaryocyte population. Megakaryocytes were then purified using a discontinuous BSA gradient and plated on 100 μg/mL immobilized fibrinogen for 3 hours at 37°C. Images were captured using a Nikon E1000 microscope at 200X magnification, and the number of proplatelet-producing megakaryocytes were quantified.

### Western blotting

To expand the megakaryocyte compartment bone marrow from PKCε^-/-^ and their corresponding WT littermate control mice was cultured in IMDM with FBS, P/S, and 50 ng/mL TPO for 5 days. Megakaryocytes were isolated using a discontinuous BSA gradient as previously described [24]. The resulting megakaryocytes were then treated with 50 ng/mL TPO or ddH2O for 10 minutes at 370C. After TPO treatment the megakaryocytes were lysed using 2X Laemmeli buffer and the DNA was sheared using a 27-gauge needle. The samples were then boiled for 5 minutes prior to resolving via SDS-PAGE. After the proteins were transferred to nitrocellulose membrane, the membranes were probed using an antibody to the protein of interest at a 1:1000 concentration. The membranes were imaged using a Li-Cor (Lincoln, NE) Odyssey infrared imager.

### Statistics

In each case a student’s T-test (2-tailed, unpaired) was used to compare PKCε^-/-^ values to PKCε^+/+^ values. A p-value of less than 0.05 was considered significant. Graphs depict mean values with SEM for all data sets. Individual data points can be found to the left of each bar graph.

## Results

### PKCε^-/-^ mice have increased platelet mass

**Fig 1:**
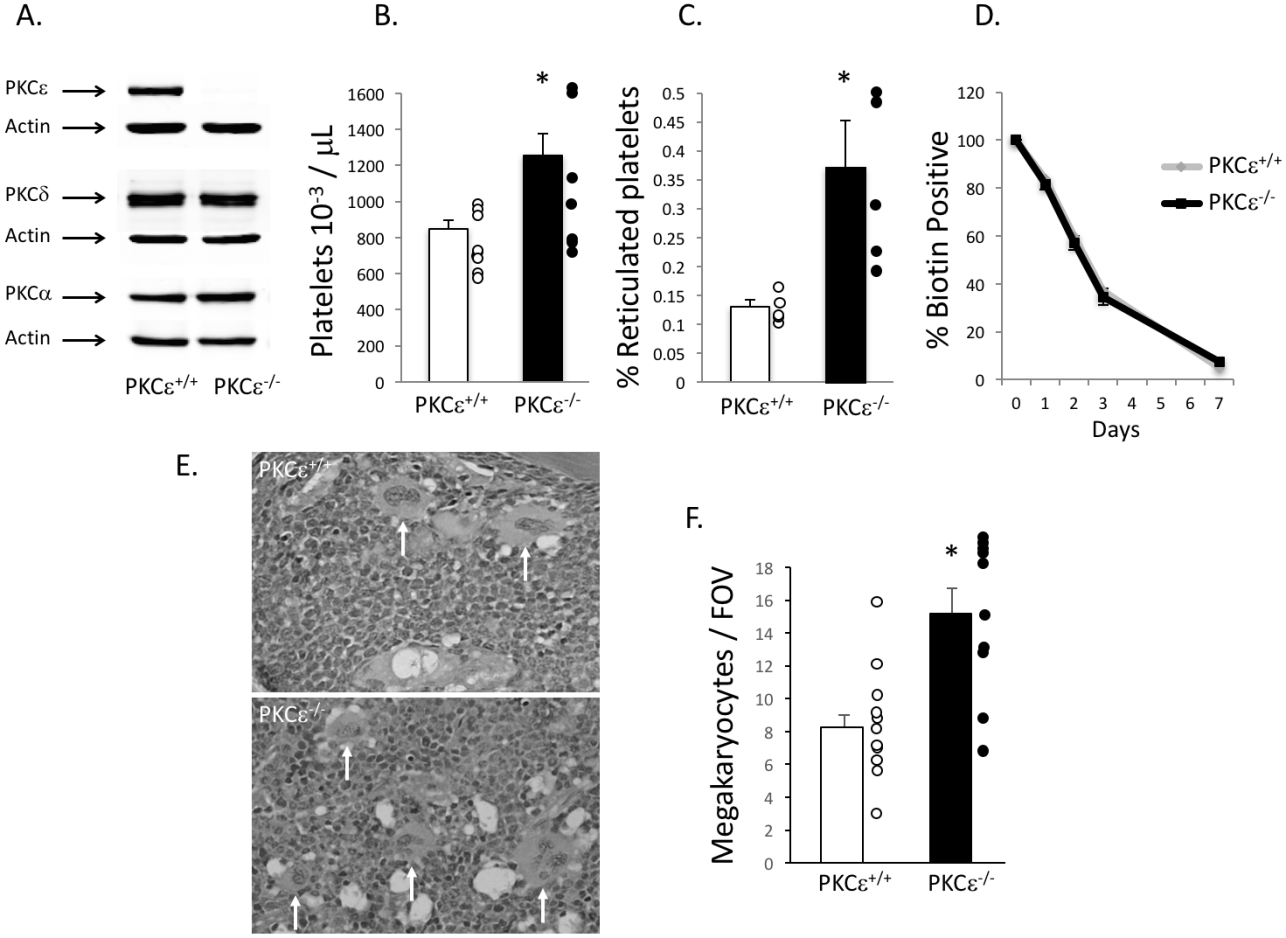
Platelet mass is enhanced in PKCε deficient mice. A) Western blot analysis of PKCε, PKCα, and PKCδ in PKCε^+/+^ and PKCε^-/-^ mouse megakaryocytes (n = 3). Actin was used to assess loading. B) Platelet counts from PKCε^-/-^ and WT littermate control (PKCε^+/+^) whole blood (n = 9). C) Reticulated “new” platelets expressed as a percent of total blood cells in PKCε^+/+^ and PKCε^-/-^ mice (n = 6). D) Platelet clearance in PKCε^+/+^ and PKCε^-/-^ mice (n = 3). E) Representative images of femur sections stained with H&E. White arrows denote megakaryocytes. Images were captures used a Nikon E1000 microscope at 200X magnification. F) Quantitation of megakaryocytes per field of view (FOV). * p < 0.05, n = 11.

To elucidate the role of PKCε in megakaryocyte differentiation and platelet production, we used a PKCε^-/-^ mouse model. We confirmed that PKCε was indeed absent in megakaryocytes from PKCε^-/-^ mice, and that protein expression of several other PKC isoforms was unaltered in megakaryocytes with PKCε deficiency (Fig 1A). Initial blood draws revealed that most blood cell counts were not different from WT littermate control mice (Table 1). However, this was not true for platelet counts as PKCε^-/-^ had significantly more circulating platelets than their WT counterparts (Fig 1B). The increase in platelet count was due to increased platelet production as PKCε^-/-^ mice had more reticulated platelets (Fig 1C), while platelet clearance was unaltered (Fig 1D). Furthermore, after sectioning and staining femurs from PKCε^+/+^ and PKCε^-/-^ mice with hematoxylin and eosin (H&E), we noted that PKCε^-/-^ mice had more megakaryocytes per FOV (Fig 1E and 1F), which is consistent with the observed increase in platelet count in PKCε^-/-^ mice.

**Table 1:** Blood cell counts in PKCε^+/+^ and PKCε^-/-^ mice.

Parameter	PKCε^+/+^	PKCε^-/-^	p-value
WBC (10^3^/μL)	8.59 ± 0.96	10.78 ± 1.22	0.169
LY (10^3^/μL)	6.28 ± 0.59	7.59 ± 0.83	0.199
Hct (%)	55.30 ± 3.19	52.69 ± 3.45	0.589
RBC (10^6^/μL)	11.20 ± 0.51	11.17 ± 0.66	0.953
MPV (fL)	4.65 ± 0.10	4.53 ± 0.11	0.437

Hematologic parameters for PKCε^-/-^ and WT littermate control mice (PKCε^+/+^) collected using whole blood. WBC = White Blood Cell, LY = Lymphocyte, Hct = Hematocrit, RBC = Red Blood Cell, MPV = Mean Platelet Volume.

### Bone marrow progenitor cell distribution is altered in PKCε^-/-^ mice

**Fig 2:**
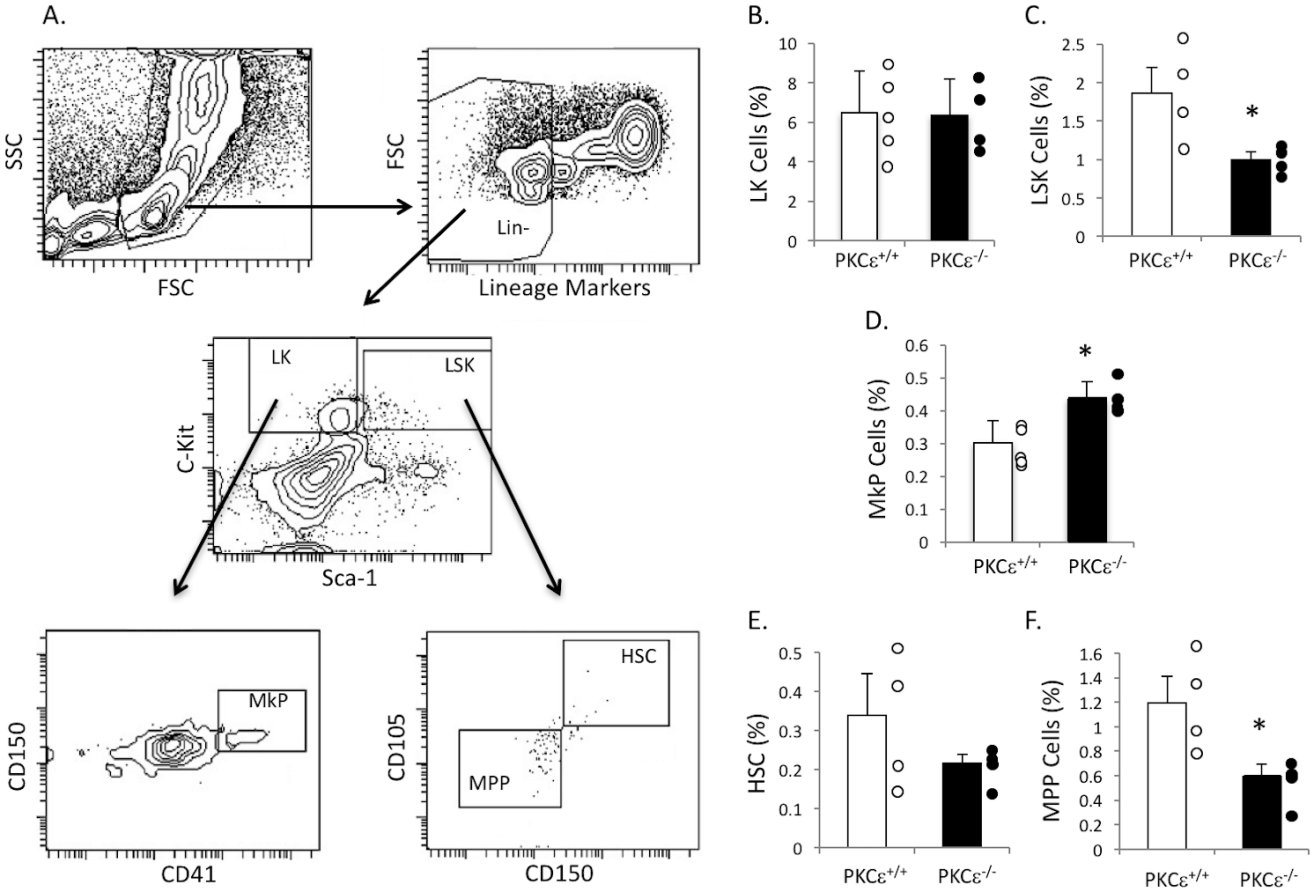
PKCε^-/-^ mice have a reduced LSK population, but a heightened megakaryocyte progenitor cell population. A) Schematic showing gates used to define each progenitor cell population. LK cells are defined as Lineage (Lin) negative cells that stain C-Kit+, Sca-1-. LSK cells stain Lin-, Sca-1+, C-Kit+. Megakaryocyte progenitors (MkP) are from the LK population and stain CD41+, CD150+. The LSK population is used to define multipotent progenitor cells (MPP), which stain CD105- and CD150-, as well as hematopoietic stem cells (HSC), which stain CD105+ and CD150+. B-F) Quantification of each progenitor cell population as defined in A in PKCε^+/+^ and PKCε^-/-^ mouse bone marrow expressed as a percentage of total bone marrow cells. * p < 0.05 compared to PKCε^+/+^, n = 4.

Platelets are produced by megakaryocytes, which arise from progenitor cells in the bone marrow. Therefore, we analyzed bone marrow from PKCε^-/-^ and WT littermate control mice for different progenitor cell populations using flow cytometry. Hematopoietic lineage negative, Sca-1 negative, c-Kit positive (LK) populations were similar in both the PKCε^-/-^ and WT littermate control bone marrow (Fig 2B). However, the lineage negative, Sca-1 positive, c-Kit positive (LSK) population was decreased in PKCε^-/-^ mice compared to WT littermate control (Fig 2C). Conversely, the megakaryocyte progenitor cell population (LK, CD150+, CD41+) was significantly enhanced in PKCε deficient bone marrow (Fig 2D). Furthermore, the multipotent progenitor cell population (LSK, CD105-, CD150-) was also decreased in PKCε^-/-^ mouse bone marrow (Fig 2E), while the hematopoietic stem cell population (LSK, CD105+, CD150+) was not altered (Fig 2F). These data indicate a shift in PKCε^-/-^ bone marrow in favor of megakaryocyte progenitor cell production.

### Megakaryocyte differentiation is enhanced with exogenous TPO

**Fig 3:**
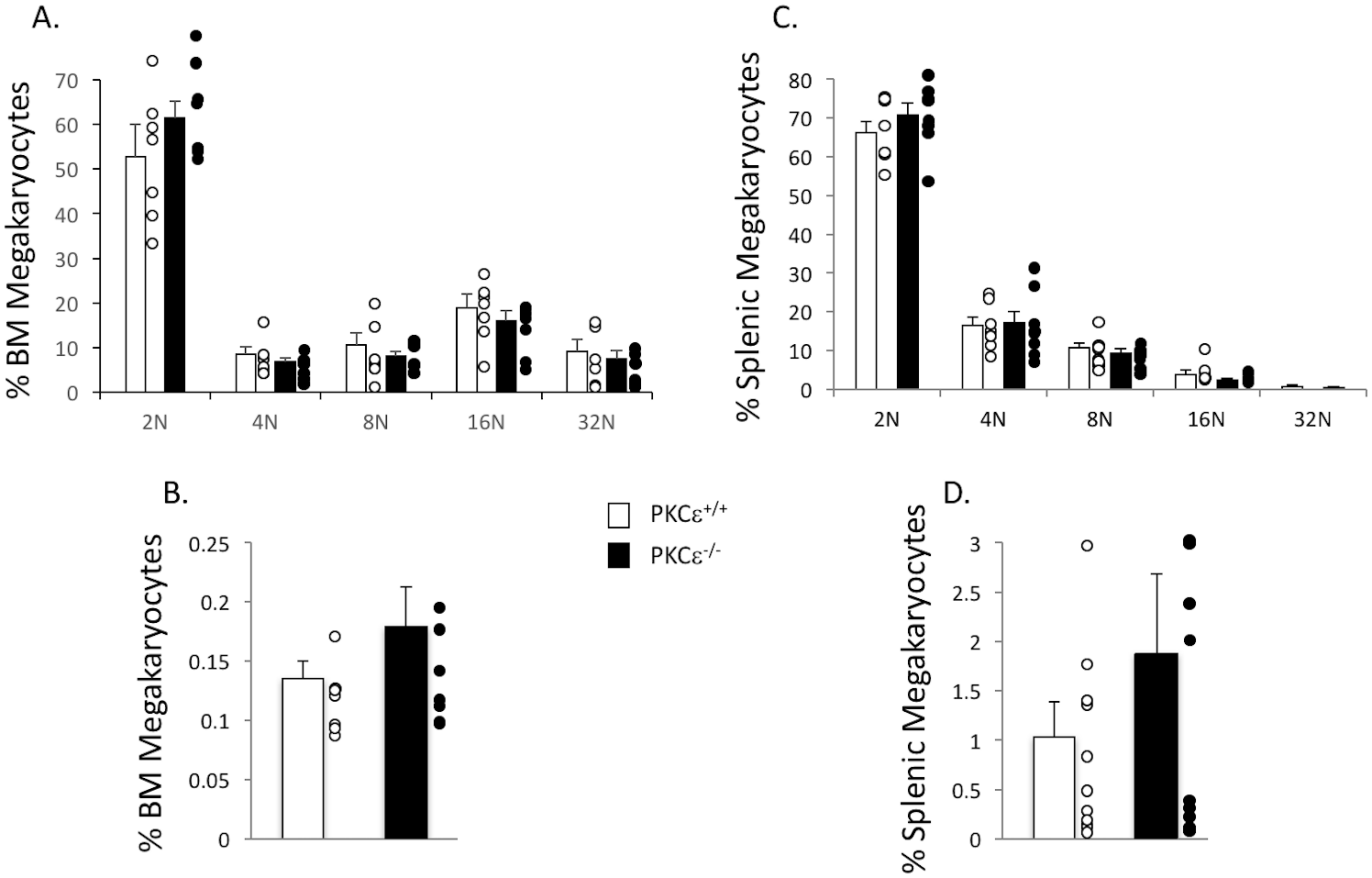
Bone marrow and splenic megakaryocyte number and DNA content are unaltered in PKCε^-/-^ mice. A) Quantification of bone marrow megakaryocyte DNA content expressed as a percentage of total megakaryocytes (n = 7). B) Quantification of bone marrow megakaryocyte number expressed as percent nucleated bone marrow cells (n = 7). C) Quantification of splenic megakaryocyte DNA content expressed as a percentage of total megakaryocytes (n = 8). D) Quantification of splenic megakaryocyte number expressed as a percentage of nucleated spleen cells (n = 8).

**Fig 4:**
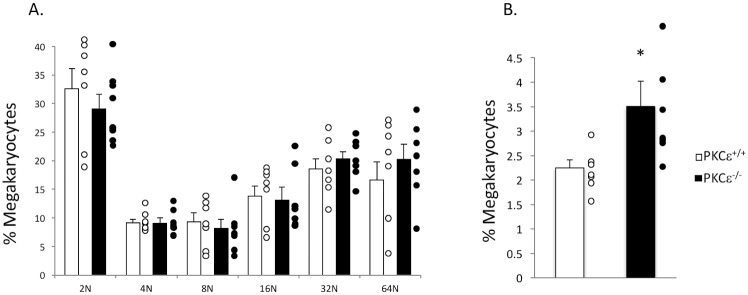
Megakaryocyte number is enhanced in PKCε^-/-^ bone marrow cultured with exogenous TPO. A) Megakaryocyte DNA content in PKCε^-/-^ and PKCε^+/+^ bone marrow cultured in the presence of 50 ng/mL TPO. B) Megakaryocyte number is cultured bone marrow from PKCε^-/-^ and PKCε^+/+^ mice supplemented with 50 ng/mL TPO. * p < 0.05 compared to PKCε^+/+^, n = 7.

Because we noted an increase in platelet production and an increased megakaryocyte progenitor cell number in PKCε^-/-^ bone marrow, we analyzed bone marrow and spleen from PKCε^-/-^ and WT littermate control mice to determine if megakaryopoiesis is altered in these compartments. Surprisingly, there was no difference in either DNA content or the percentage of megakaryocytes in either the bone marrow or spleen of PKCε^-/-^ mice compared to WT littermate controls (Fig 3A and 3B). Similarly, PKCε^-/-^ spleen weight/body weight (g) ratio was not altered (0.00367 ± 0.00032 PKCε^+/+^ vs 0.00368 ± 0.00031 PKCε^-/-^). However, after culture of PKCε^-/-^ and WT littermate bone marrow in the presence of exogenous TPO the number of megakaryocytes in the PKCε^-/-^ cultures was significantly enhanced although megakaryocyte DNA content was unaltered (Fig 4A and 4B). These data suggest that PKCε may influence TPO signaling.

### TPO-mediated signaling is enhanced in PKCε^-/-^ megakaryocytes

**Fig 5:**
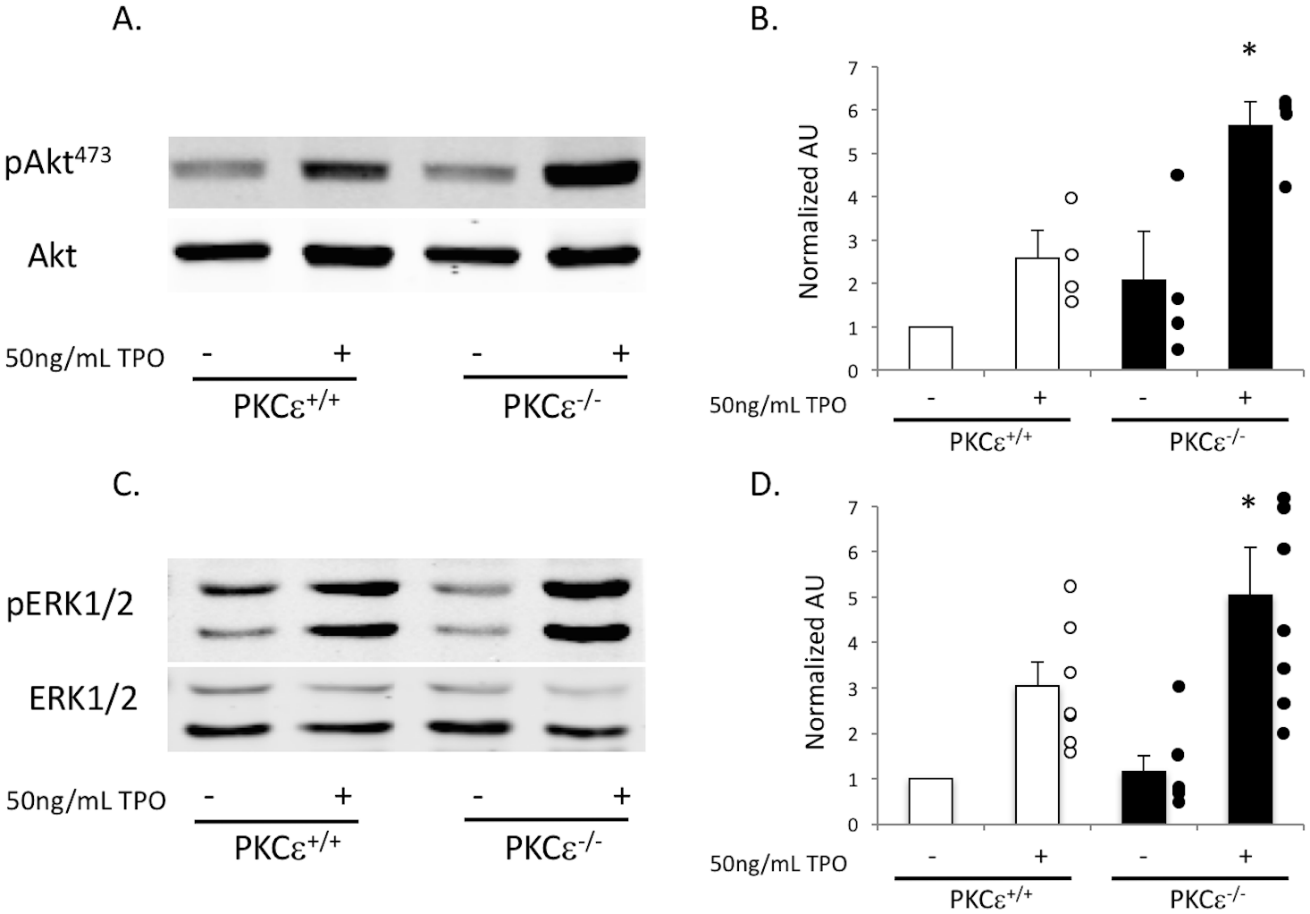
TPO-mediated Akt and ERK1/2 phosphorylation is enhanced in PKCε^-/-^ megakaryocytes. A) Western blot showing Akt phosphorylation at Ser473 in lysates from megakaryocytes isolated from PKCε^+/+^ or PKCε^-/-^ mice treated with 50 ng/mL TPO (+) or not (-). Lane loading was assessed using total Akt (n = 4). B) Quantification from A expressed as a ratio of phosphorylated Akt to total Akt and normalized to PKCε^+/+^ without TPO treatment. C) Western blot showing ERK1/2 phosphorylation in PKCε^+/+^ and PKCε^-/-^ megakaryocytes treated with or without TPO. Lane loading was assessed using total ERK (n = 7). D) Quantification of C expressed as a ratio of phosphorylated ERK1/2 to total ERK1/2 and normalized to PKCε^+/+^ without TPO treatment. * p < 0.05 compared to PKCε^+/+^ with TPO treatment.

To determine if TPO signaling is altered in megakaryocytes deficient in PKCε, we expanded megakaryocytes in culture and isolated them using a discontinuous BSA gradient. We then treated the megakaryocytes with 50 ng/mL TPO for 10 minutes at 37°C. Western blot analysis of megakaryocyte lysates revealed that TPO-mediated signaling was enhanced, as we observed a statistically significant increase in phosphorylated Akt and ERK1/2 in PKCε^-/-^ mouse megakaryocytes compared to littermate control (Fig 5A–5D). However, upstream signaling appeared unaltered, as we noted no difference in JAK2 or FAK Y925 phosphorylation in PKCε^-/-^ megakaryocytes compared to control (data not shown). This suggests that PKCε regulates TPO signaling downstream of JAK2 and FAK, but upstream of Akt and ERK1/2.

### PKCε^-/-^ mice recover faster from immune-induced thrombocytopenia than littermate control mice

**Fig 6:**
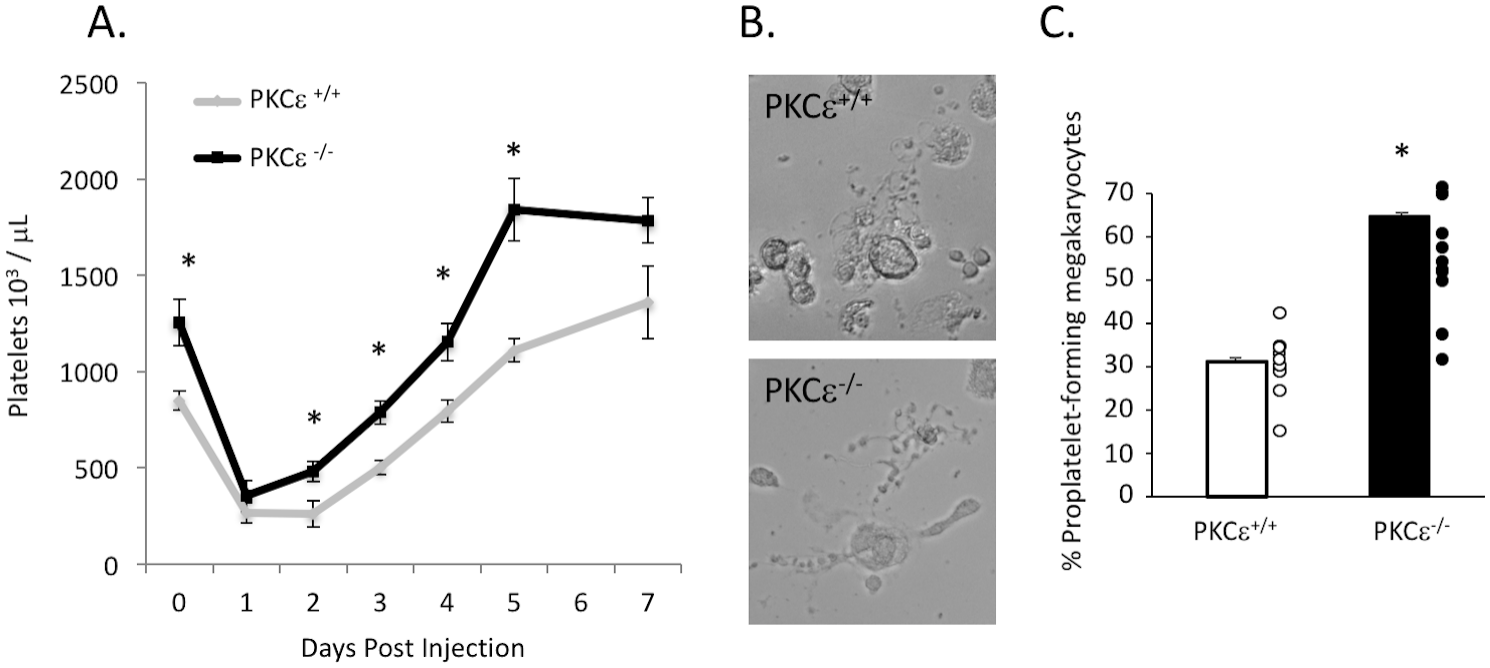
Platelet recovery and rebound thrombocytosis is enhanced in PKCε^-/-^ mice following immune-induced thrombocytopenia. A) PKCε^+/+^ and PKCε^-/-^ Mice were injected (I.P.) with 50 μg/kg anti-mouse CD41 antibody at day 0. Blood was collected daily via submandibular puncture for 5 days and again on day 7 and platelets were enumerated. * p < 0.05 compared to corresponding PKCε^+/+^ time point, n = 7. B) Representative images of proplatelet producing megakayrocytes. C) Quantitation of proplatelet production taken from several fields per experiment, expressed as a percentage of total megakaryocytes. *p < 0.05, n = 11.

PKCε^-/-^ and WT littermate control mice aged 10–12 weeks were given anti-mouse CD41 antibody via I.P. injection to cause thrombocytopenia. Their platelet counts were monitored daily for 7 days. After 24 hours, platelet counts began to rise in PKCε^-/-^ mice, while this process did not occur in control mice until after 48 hours. From that point PKCε^-/-^ mice had a higher rate of recovery than littermate control mice, as well as a heightened rebound thrombocytosis (Fig 6). These data suggest that PKCε^-/-^ mice recover faster from thrombocytopenia than control mice, and is consistent with the enhanced number of megakaryocytes observed in the bone marrow of PKCε^-/-^ mice. Additionally, we determined whether or not proplatelet production is enhanced in PKCε knockout mice, since it has previously been reported that PKCε deletion inhibits proplatelet production [25]. Conversely, we show that megakaryocytes from PKCε^-/-^ mice plated on immobilized fibrinogen produce more proplatelets than WT control megakaryocytes. These data support our observation that platelet recovery following immune thrombocytopenia is enhanced in PKCε^-/-^ mice.

## Discussion

In this report we use a PKCε knockout mouse model to show that PKCε is a negative regulator of megakaryopoiesis. We show that PKCε^-/-^ mice have a greater platelet mass than littermate control mice due to enhanced platelet production, as well as enhanced proplatelet production *in vitro*. Furthermore, several progenitor cell populations in the bone marrow are altered with PKCε deficiency. PKCε^-/-^ mice have reduced LSK, HSC, and MPP populations, but an enhanced MkP population. Additionally, bone marrow from PKCε^-/-^ mice cultured in the presence of exogenous TPO produces more megakaryocytes than littermate control bone marrow. Not surprisingly, TPO-mediated signaling is also enhanced as both Akt and ERK1/2 phosphorylation is elevated in PKCε deficient megakaryocytes. Finally, PKCε^-/-^ mice recover faster from immune-induced thrombocytopenia and have an enhanced rebound thrombocytopenia compared to their WT littermates in part due to enhanced proplatelet production. The above data strongly suggest that PKCε is a negative regulator of megakaryopoiesis.

PKC’s have long been implicated in megakaryocyte differentiation. Recently, we, and others, reported on several specific PKC isoforms using knockout mouse models. We revealed that PKCδ is a negative regulator of megakaryopoiesis, while PKCθ is dispensable [24, 26]. Williams et al., showed that PKCα is also a negative regulator of megakaryopoiesis [27]. Another recent report from Machlus et al, suggests that a PKC substrate, myristolated alanine-rich C-kinase substrate (MARCKS) also negatively regulates megakaryocyte maturation and proplatelet formation [28]. Here we show that PKCε is also a negative regulator of megakaryopoiesis. Therefore, PKC’s have a prominent role in megakaryocyte differentiation and proplatelet production.

Expression of PKCε in human megakaryocytes differs from that of mouse megakaryocytes. In human megakaryocytes, PKCε expression increases early during differentiation, but decreases as megakaryocytes prepare for platelet release [21]. In mice PKCε expression is high in mature megakaryocytes (Fig 1). While this may seem problematic, data collected using CD34+ human cells from patients with either primary myelofibrosis (PMF) or essential thrombocythemia (ET) appears consistent with findings reported in this manuscript. CD34+ cells from patients with PMF cultured to induce megakaryocyte differentiation have heightened PKCε expression, while CD34+ cells from patients with ET cultured to induce megakaryocyte differentiation have reduced PKCε expression compared to control [29]. This is consistent with our murine data in that cultures from PMF patients (high PKCε expression) have reduced megakaryocyte differentiation, while cultures from ET patients (low PKCε expression) have robust megakaryocyte differentiation [25, 30]. Therefore, reduced PKCε expression in human CD34+ cells results in enhanced megakaryocyte differentiation.

PKCε regulates differentiation of a number of different cell types [31–33]. Specifically, PKCε regulates human pluripotent stem cell (hPS) self-renewal as inhibition of PKCε via siRNA reduced fibroblast growth factor-2 mediated signaling, which is essential for hPS self-renewal [31]. Further, using bone marrow from 5-FU treated mice, Shiroshita et al determined that PKCε was expressed in Lin- cells and that inhibition of PKCε using a peptide inhibitor reduced LK and LSK populations *in vitro* [34]. These data are consistent with our data concerning the LSK population. However, we did not see any alteration in the LK population in PKCε^-/-^ mice. This could be due to the cytokine cocktail they used which contained interleukin-3 and TPO only as opposed to our *in vivo* setting in which the marrow is exposed to many different factors. We also observed that other progenitor cell populations such as HSC and MPP were reduced while the MkP cell population was enhanced in PKCε^-/-^ mice. This suggests that PKCε deficiency favors megakaryocyte differentiation.

In addition to its effects on progenitor cell production, we also observed that PKCε is an important negative regulator of TPO-mediated signaling. We show that PKCε^-/-^ mouse megakaryocytes have enhanced Akt and ERK1/2 phosphorylation in response to TPO. When we explored the signaling upstream of ERK1/2 and Akt we found that JAK2 and FAK were unaltered, suggesting that PKCε functions upstream of Akt and ERK1/2, but downstream of JAK2 and FAK.

This report is in contrast to a previously published manuscript in which the authors report that PKCε is necessary for proplatelet production from mouse fetal liver-derived megakaryocytes [25]. The authors used shRNA infection of fetal liver cells to produce PKCε null megakaryocytes *in vitro*. Megakaryocytes that were devoid of PKCε either failed to produce, or produced fewer megakaryocytes. Conversely, we present data in this manuscript that shows proplatelet production is enhanced in bone marrow-derived PKCε^-/-^ megakaryocytes. The exact reason for this discrepancy is not clear, though the differences between *in vivo* and *in vitro* techniques, as well as fetal liver cells and bone marrow cells is a likely starting place.

In summary, we conclude that PKCε is a negative regulator of megakaryopoiesis and differentially regulates progenitor cell production. PKCε deficiency caused a shift towards megakaryocyte progenitor cell production that resulted in enhanced recovery from thrombocytopenia. Therefore, PKCε may be a viable therapeutic target for thrombocytopenia.

## Supporting information

S1 File NC3Rs ARRIVE guidelines checklist. (PDF)
